# High Prevalence and Genetic Heterogeneity of Genotype 3 Hepatitis E Virus in Wild Boar in Umbria, Central Italy

**DOI:** 10.1155/2023/3126419

**Published:** 2023-06-30

**Authors:** Farzad Beikpour, Monica Borghi, Eleonora Scoccia, Teresa Vicenza, Andrea Valiani, Simona Di Pasquale, Silvia Bozza, Barbara Camilloni, Loredana Cozzi, Piero Macellari, Vito Martella, Elisabetta Suffredini, Silvana Farneti

**Affiliations:** ^1^Department of Veterinary Medicine, University of Bari Aldo Moro, Bari, Italy; ^2^Department of Food Safety, Nutrition and Veterinary Public Health, Istituto Superiore di Sanità, Rome, Italy; ^3^Istituto Zooprofilattico Sperimentale dell'Umbria e delle Marche, Perugia, Italy; ^4^Microbiology and Clinical Microbiology Section, Department of Medicine and Surgery, University of Perugia, Perugia, Italy; ^5^Sanità Veterinaria e Sicurezza Alimentare-Sezione Sanità Pubblica Veterinaria, Regione Umbria, Direzione Salute e Welfare-Servizio Prevenzione, Perugia, Italy

## Abstract

Hepatitis E virus (HEV) is an important human pathogen and, in developed countries, most human infections are due to a zoonotic cycle, mainly maintained by domestic and wild suids. In European countries several genotype 3 strains have been found to circulate in human population and animal reservoirs, with human infections being related mostly to pork or wild boar meat consumption. In this study, we surveyed HEV circulation in wild boar in Umbria (Italy) during the 2021–2022 hunting seasons, using a stratified sampling. Liver samples were tested for HEV presence by real-time RT-qPCR. Positive samples were characterized by nested RT-PCR followed by sequencing of partial region of the capsid gene. Overall, 78 out of 179 wild boar liver samples tested positive to HEV (43.6%), with viral load ranging between 1.47 and 7.35 log genome copies/g (median 3.20 log). Variations, although not statistically significant, were observed considering geographical, age, weight, and gender factors, in terms of either prevalence or viral load. In particular, in animals younger than 1 year of age, the viral load was 2 log higher (median viral load of 5.50 log genome copies/g of liver tissue) than in older age groups. Sequence analysis characterized the 41 obtained sequences into genotype 3 subtype 3c (*n* = 4), 3f (*n* = 11), and 3e (*n* = 1), while 13 sequences clustered with two genotype 3 genomes (GenBank MF959764 and MK390971) still unassigned to subtypes. Additional, 12 sequences did not cluster with any known subtype or unassigned genome and on phylogenetic analysis segregated into two distinct groups of eight and four sequences, respectively. Interestingly, some wild boar sequences of subtype 3f were intermingled with sequences of HEV strains previously identified in human patients in Central Italy. Sharing of molecular data for HEV in animals is pivotal to decipher the intricate ecology of HEV.

## 1. Introduction

Human hepatitis E, an emerging foodborne disease, is caused by human hepatitis E virus (HEV), recently classified as species *Paslahepevirus balayani* (formerly Orthohepevirus A), a member of the family Hepeviridae. The species *P. balayani* incorporates at least eight distinct genotypes, which are further subtyped on the basis of phylogenetic analysis of the HEV ORF2 genome fragment (capsid region). Genotype 3 viruses, with the most common subtypes HEV-3c and HEV-3e, f, g, appear to represent the main source of zoonotic transmission of HEV in Europe so far, but several clades of unidentified HEV strains have been emerging recently [1–3].

In most cases, HEV infection remains asymptomatic or becomes clinically evident as a form of acute self-limiting hepatitis that predominantly affects male individuals over 50 years of age [4–6]. Symptoms generally are self-limiting and include nausea, vomiting, abdominal pain, asthenia, lack of appetite, and jaundice in the acute forms, although HEV infection may be a significant cause of morbidity and mortality in immunocompromised individuals and pregnant women [5, 7, 8]. In Europe, the number of confirmed HEV infection has increased over the last 10 years, showing a 10-fold increase in the period 2005–2015 and, according to European Food Safety Authority (EFSA), the main source of HEV transmission is the consumption of contaminated food [5, 9].

In Italy, one of the main risk factors for HEV infection is represented by the regional consumption habits, such as raw or undercooked wild boar meat, particularly liver-based meat products, sausages, and salami [10–12]. These products are widely consumed in Central Italy where it has been reported that more than 40% of sera from blood donors were positive for the presence of anti-HEV antibodies [13–15]. The interest in the epidemiology of HEV infection has increased in the recent years and studies carried out in Umbria, a region of the Central Italy, have reported an HEV seroprevalence of 10.3% [16].

Following this evidence and together with a likely increase in clinicians' awareness, a steady increase in human cases of HEV infection has been reported in Umbria starting from February 2020. During the first half of the 2022, a total of eight cases of PCR-confirmed HEV infection were described and one patient developed severe symptoms resulting in death (Bozza & Camilloni, unpublished). Significantly, molecular characterization of viral RNA isolated from the fecal samples of two patients and the suspected food vehicle correlated these two cases of acute hepatitis E to the consumption of wild boar meat (Farneti, unpublished).

Based on these premises and considering the substantial lack of information on the HEV strains circulating in wild boars in Umbria, the health authorities of Umbria Region established an active surveillance plan to gather information on the epidemiology of HEV infection in the wild boar population of the region during the hunting season from October 2021 to January 2022. Thus, the aim of the present study was to investigate HEV prevalence in wild boars in Umbria and to provide molecular typing of the detected viral strains in order to improve the knowledge on HEV circulation and better address the public health issue associated to foodborne transmission to the population.

## 2. Materials and Methods

### 2.1. Sampling

**Figure 1:**
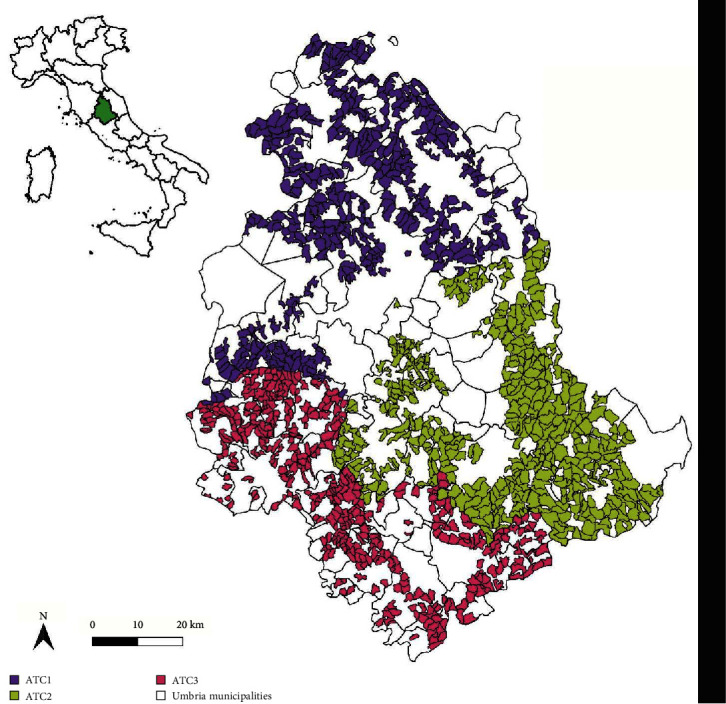
Geographic distribution of the three different hunting districts (ATCs) in the region of Umbria.

In order to estimate the prevalence of HEV in wild boars (*Sus scrofa*) in Umbria, a region of Central Italy, during the 2021–2022 hunting seasons, a specific sampling plan was developed taking into account the surface of the hunting territory, comprising the three hunting districts (“Ambiti Territoriali Caccia”, ATC) and the duration of the monitoring activities. For sampling plan design, data on population were extracted from previous hunting seasons (19,248 animals shot during 2019–2020) and, since no specific data on HEV prevalence in the study area were available in literature, a 20% prevalence was hypothesized. This led to a sample size of 170 animals (95% confidence level, 6% precision) that were then proportionally distributed across the different ATCs (Figure 1) based on their geographical extension and evenly distributed over the sampling months, spanning the period from October 2021 to January 2022.

Collection of liver samples from wild boar was performed by teams of hunters and veterinarians actively engaged in the implementation of the sampling plan. For each hunted animal, a sampling report was filled, including the characteristics of the animal and of the sample (date of sampling, ATC, sex, presumed age, weight); only the samples matching the selection criteria defined in the sampling design (ATC of origin) were subjected to analysis. Samples (approximately 150 g of liver tissue and, when possible, 20 g of muscle tissue) were taken using sterile instruments. Occasionally, muscle tissue from different animals shot in the same hunting trip was collected together. Samples were transported at a controlled temperature (+4°C) to the laboratory, where they were processed immediately or stored at –20°C until analysis was performed.

### 2.2. Sample Preparation

Liver sample preparation was performed as previously described [17]. In short, 5 g of chopped liver tissue was added to a 50 ml falcon tube containing 3 mm sterile glass beads. Following the addition of 10 *μ*l of process control virus (Mengovirus, strain MC_0_, ∼10^5^ TDCI_50_/ml), each sample was treated with 7 ml of peqGOLD TriFast™ (VWR Chemicals, PA, USA) and vortexed at high speed for 2 min to completely homogenize the tissue. Samples were then centrifuged at 8,000 × *g* for 20 min at 4°C, and the recovered supernatant was mixed with chloroform (0.2 v/v), briefly vortexed and incubated at the room temperature for 15 min. Finally, samples were centrifuged again at 8,000 × *g* for 20 min at 4°C and the aqueous solution was recovered and measured. Available muscle samples were processed with the same procedure and were tested only in presence of HEV detection in liver tissue of the corresponding animal.

Viral RNA extraction was carried out on 1 ml of the aqueous solution, using the NucliSENS miniMag extraction system (bioMérieux, France) according to the manufacturer's instructions. RNA was eluted in 100 *μ*l and stored at –80°C until molecular assay. A negative extraction control (molecular grade water) was included in each extraction session to rule out contamination.

### 2.3. Real-Time RT-qPCR

Detection of HEV viral RNA was performed by real-time RT-qPCR as previously described [17]. Reactions were prepared in a total volume of 25 *μ*l, using the RNA UltraSense™ One-Step RT-qPCR System (Life Technologies, CA, USA), 5 *μ*l of the sample nucleic acids, 1.25 *μ*l of enzyme mix, reverse primer (900 nM), forward primer (500 nM), and probe (250 nM) (Table 1). The thermal profile included reverse transcription at 50°C for 60 min; reverse transcriptase inactivation at 95°C for 5 min; and 45 cycles of 15 s at 95°C, 1 min at 60°C, and 1 min at 65°C for the amplification. Analyses were done in duplicate on a QuantStudio 12 K Flex (Applied Biosystems, CA, USA), and the mean concentration of the two replicate reactions was applied for quantification. An in vitro synthesized (Eurofins Genomics, Germany) linearized DNA containing the target sequence of the real time, quantified by Qubit mesurement (Thermo Fisher Scientific, MA, USA), was used to generate the standard curve (range: 10^5^–10^0^ genome copies/*μ*l). Only curves with a slope between –3.1 and –3.6 and an *R*^2^ ≥0.98 were used for quantification.

For quality assurance purposes, a negative PCR control, a negative environmental control, and the negative extraction control (also tested in duplicate) were included to monitor contamination events. PCR inhibition was excluded using an external amplification control (*in vitro* synthesized RNA coding for the target sequence) and amplifications were accepted if inhibition was ≤50%. The analysis of the process control virus according to Costafreda et al. [23] was used to evaluate viral recovery from liver tissue, and the exaction efficiency was considered acceptable for values ≥1%.

### 2.4. Nested RT-PCR

To genotype and subtype those samples that were positive for HEV by RT-qPCR, an nested RT-PCR was carried out on the ORF2 region of the HEV genome. Primers HE044 and HE040 [20], providing a 506 bp amplicon, were used for the first PCR, and primers HE110-2-mod and HE041 [21, 22], amplifying a 467 bp fragment of the ORF2 region (Table 1), were used for the nested PCR.

The RT-PCR was carried out in a 25 *μ*l reaction volume, using the Superscript IV One-Step RT-PCR System (Thermo Fisher Scientific, Waltham, MA, USA), 5 *μ*l of sample RNA, and primers 0.4 *μ*M. The RT-PCR was run as follows: reverse transcription at 45°C for 10 min, inactivation for 2 min at 98°C, followed by 40 cycles of 98°C for 10 s, 53°C for 20 s, 72°C for 30 s, and a final step of 72°C for 5 min. Afterward, 2 *μ*l of the product from the first PCR was used as a template in the nested PCR, using DreamTaq DNA polymerase enzyme (Thermo Fisher Scientific) and the following thermal profile: 2 min at 95°C, 45 cycles of 95°C for 30 s, 53°C for 30 s, 72°C for 45 s, and a final step of 72°C for 7 min. As a PCR positive control, RNA extracted from the HEV strain 47832c grown on A549/D3 cell line [24] was used. The amplifications were carried out in a PT-200 Thermal Cycler (MJ Research, MA, USA), and the nested PCR products were observed on gel electrophoresis (1.5% agarose gel), stained with GelRed (Biotium, CA, USA).

### 2.5. Sequencing and Phylogenetic Analysis

Purification of PCR products was done using the GRS Full Sample Purification Kit (GRiSP, Porto, Portugal), and samples were submitted to sequencing on both strands (Bio-Fab Research, Italy). Consensus sequences were assembled using BioEdit software (version 7.2.5) and were submitted to BLAST analysis and to the HEV typing tool (http://www.rivm.nl/mpf/typingtool/hev/, last accession on May 9, 2023) for genotyping.

Phylogenetic relationships were analyzed using the maximum likelihood method and Tamura–Nei model [25] on MEGAX software [26]. The tree reliability was assessed through bootstrap analysis (1,000 replicates). The analysis also included 32 representative sequences of human (hu), swine (sw), wild boar (wb), and food (food) origin downloaded from GenBank; sequences were selected based on geographic origin, with a priority to sequences from Italy. The HEV partial ORF2 nucleotide sequences were submitted to NCBI GenBank under the accession numbers OQ349517–OQ349557.

### 2.6. Statistical Analysis

The 95% confidence interval was calculated for proportions. For the analysis of viral loads, the median of the quantifiable values (i.e., results above the limit of quantification of the real-time RT-PCR, 1.40 genome copies (g.c.)/*μ*l of RNA), the minimum, maximum, and the quartiles were calculated. The *χ*^2^ test and one-way ANOVA were performed, respectively, to compare prevalence values and viral loads in the different groups. Statistical calculations were done using MedCalc Statistical Software v18 (MedCalc Software bvba, Ostend, Belgium).

## 3. Results and Discussion

### 3.1. HEV Prevalence and Viral Loads in Wild Boar Livers

Of the 179 wild boar liver samples tested in the study, 78 (43.6%, 95% CI: 36.5–50.9) were positive for HEV (Table 2). Viral concentration in the 53 quantifiable samples ranged between 1.47 and 7.35 log g.c./g (median 3.20 log). HEV prevalence in liver samples was variable according to hunting area, with a 50.0% positivity rate (95% CI: 26.6–50.3) in wild boar sampled in ACT2 (southeast of Umbria region), 40.5%, 95% CI: 39.0–61.0 in southwest (ATC3), and 37.7%, 95% CI: 27.0–55.5 in the northern part of the region (ATC1). This result is particularly significant, as it represents one of the highest prevalence values reported for HEV in wild boar, which, according to a recent systematic review and meta-analysis, range from 0% to 34% in Italy and from 0% to 56% worldwide, with an overall pooled prevalence of 8% [27]. Indeed, studies performed in the last 10 years in two nearby Italian regions, Lazio and Abruzzo, located southwest and southeast of Umbria region, respectively, displayed positivity rates of 16.3% and 9.5%, respectively [17, 28]. Interestingly, specific districts—roughly corresponding to local municipalities—within the hunting areas provided positivity rates ranging from 0% to 80% (Table S1), highlighting the heterogeneity of viral distribution among wild boar herds.

**Figure 2:**
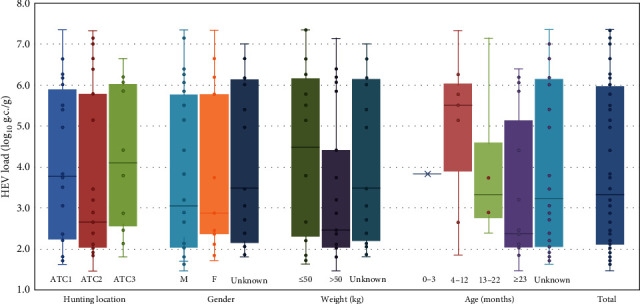
Dispersion of HEV quantitative values in liver samples according to category. The box-and-whisker plot displays median values, first and third quartiles (boxes), minimum and maximum values (whiskers), and the intermediate individual values (dots) arranged according to the different categories.

As far as age is concerned, younger animals displayed high prevalence values (50.0%, 95% CI: 15.0–85.0, for animals <3 months and 58.8%, 95% CI: 36.0–78.4, for juveniles up to 12 months of age), but HEV was also detected in a significant proportion of 1- and 2-year-old animals (37.5%, 95% CI: 21.1–57.4 and 43.1%, 95% CI: 30.5–56.7, respectively). While caution should be used in the interpretation of these data due to the applied sampling scheme, not specifically designed to assess differences among age groups, these results are in agreement with findings previously reported in similar studies in the Czech Republic [29] and in Italy [17]. Age distribution of the infection, indeed, may reflect the fact that, while in the vast majority of domesticated pigs held in swine farms, HEV infection occurs early in life and shortly after weaning [30], in wild animals (including wild boar), exposure to HEV might occur also at a later stage of the animal life, including in adults [17, 31]. Differences among the age groups were also visible for the viral loads (Table 2 and Figure 2). Animals with an estimated age between 4 and 12 months showed indeed a median viral load of 5.50 log g.c./g of liver tissue, while viral concentration was 3.32 and 2.37 log for the age classes 13–22 months and above 23 months, respectively. It is not possible to unequivocally explain the occurrence of lower median viral concentrations in older animals; however, it may be considered that HEV loads in liver vary both in the course of infection (with lower levels in the initial stages of infection and during clearance) and in relation with the immunological response of the individual that, if impaired by coinfections with other viruses, may occasionally lead to chronic persistence of HEV in swine [32, 33].

Finally, the distribution of positive results based on animal gender (52.2%, 95% CI: 40.5–63.8 and 40.4%, 95% CI: 27.6–54.7, in males and females, respectively) and weight (48.6% vs. 46.1% in animals below and above 50 kg, respectively) did not displayed evident differences. However, median viral concentrations were approximately 2 log lower in larger animals (2.46 log g.c./g of liver in animals >50 kg compared to 4.49 log g.c./g in wild boar ≤50 kg). Due to the correlation between animal weight and age in swine [34], this result may be related to the previously discussed HEV prevalence in wild boar of 12 months (or below) of age.

According to statistical analysis, none of the differences detected based on hunting location, gender, weight, or age, either in terms of prevalence or median viral loads, were statistically significant (*χ*^2^ test and one-way ANOVA *p*-value >0.05).

HEV RNA was also detected in seven of the nine muscle samples deriving from animals that had tested positive in the liver (data not shown). These samples displayed an overall low viral load, below the quantification limit for six samples and equivalent to 1.7 × 10^2^, 3.8 × 10^2^, and 1.7 × 10^3^ c.g./g in remaining three.

### 3.2. Genetic Characterization of HEV

The nested RT-PCR provided amplification of HEV partial ORF2 region in 38 of the 78 positive liver samples (48.7%) and four of the seven positive muscle samples. Attempts for the amplification of remaining samples were unsuccessful, mostly in relation to their low viral load (below 10 g.c./*μ*l of RNA). Only one sample with high viral concentration (2.5 × 10^4^ g.c./*μ*l of RNA) was not amplified despite repeated attempts, possibly in relation to mutations in primer binding sites. Among the 42 sequences, one sample (#156, corresponding to a male wild boar, aged ≥ 23 months, hunted in December 2021 in ATC2) displayed a mixed electropherogram, likely due to coinfection by two distinct HEV strains.

On sequence analysis with BLAST, all 41 sequences of the study belonged to genotype 3. Three different subtypes were detected according to the HEV typing tool: 3c (four samples), 3f (11 samples, divided in two clusters), and 3e (one sample). Thirteen samples clustered with two genomes (MF959764 and MK390971) unassigned to any subtype according to the current HEV classification [3] and were therefore reported by the typing tool as 3_uc3 (12 samples) and 3_uc6 (one sample), respectively. Finally, 12 sequences could not be assigned to any subtype nor did they cluster with any other reference genome.

**Figure 3:**
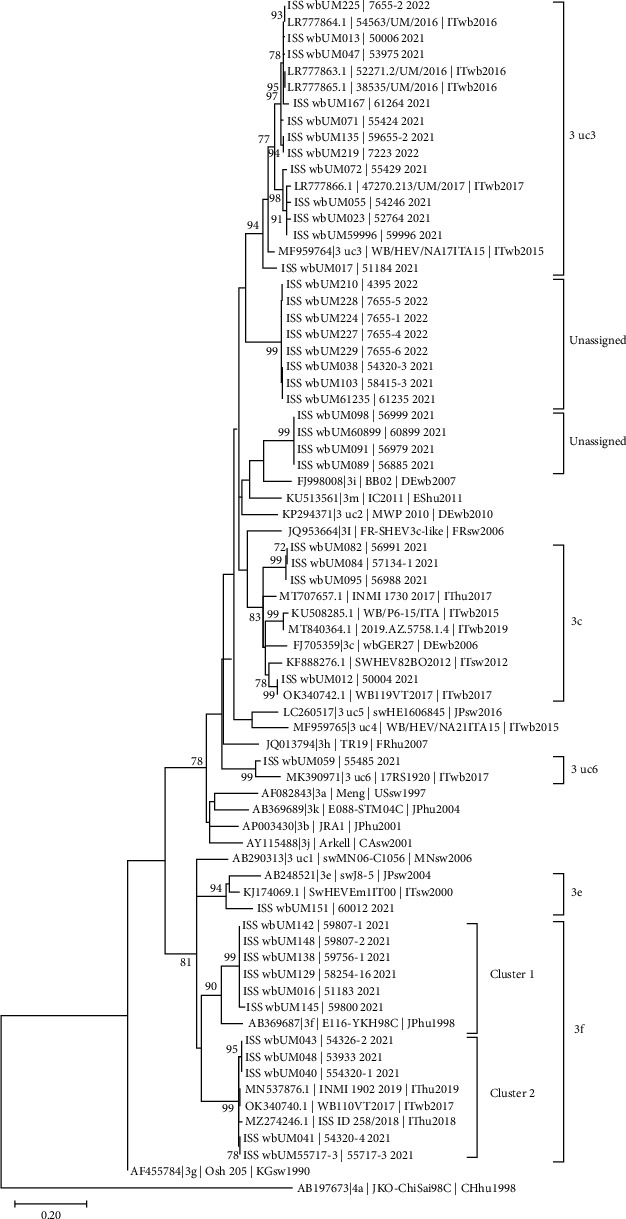
Phylogenetic tree of the partial HEV ORF2 sequences obtained from wild boar (liver and muscle samples). The maximum likelihood phylogenetic tree was constructed using the Tamura–Nei model with a discrete gamma distribution and sites evolutionarily invariable (TN93 + G + I). Codon positions included were 1st + 2nd + 3rd + noncoding. The tree is drawn to scale, with branch lengths measured in the number of substitutions per site. The analysis involved 73 nucleotide sequences and a total of 410 positions. Bootstrap values >70% are shown next to the branches. Analyses were conducted in MEGA X. Genotype and subtypes are referred according to Smith et al., 2020 [3]; genotype 3 genome sequences unassigned according to current classification (AB290313, KP294371, MF959764, LC260517, MF959765, MK390971) were reporting adding also the uc number adopted in the HEV typing tool.

Phylogenetic analysis (Figure 3) confirmed subtype classification of the HEV sequences. In particular, the 3c subtype sequences segregated within a defined cluster in the tree, with exception of one sample (wbUM012) that displayed high identity (99.8% nt) with a sequence from another wild boar hunted in 2017 in the province of Viterbo (Lazio, Italy), an area bordering with the sampling site in ATC3.

Two clusters of 3f sequences were clearly distinguishable in the tree. Cluster 1—related to the prototype strain of the 3f subtype (GenBank a.n. AB369687)—included only the six sequences from the current study, and no other sequence from Italy being available in the databases. On the opposite, in cluster two, the sequences from the present study (*n* = 5) were intermingled with sequences from two human cases of acute hepatitis E reported in Central Italy in 2018 and 2019 (MN537876 and MZ274246) and with a sequence from another wild boar hunted in the province of Viterbo in 2017. Since wild boar population may disperse on the territories regardless of geographical/administrative borders, this clearly suggests that a continuous and systematic exchange of information between local health authorities and national reference laboratories are required to trace cases and possible sources.

A large group of sequences from this study (*n* = 12) clustered in a distinct branch with one of the six G3 genomes still unassigned to any subtype in the current classification [3], GenBank MF959764, a sequence deriving from a wild boar hunted in Campania (southern Italy) in 2015. Interestingly, other four sequences from wild boar hunted in Umbria from 2016 to 2017 (LR777863 to LR777866 [35]) clustered in this branch of the tree, clearly showing that this HEV lineage has been steadily circulating in the region for years. Similarly to this group, a single sequence (ISS wbUM059) clustered with another unassigned G3 HEV genome (MK390971), a strain detected in a wild boar hunted in northeast Italy in 2017. The phylogenetic analysis provided further insight into the 12 sequences unassigned using the HEV typing tool. The unassigned sequences clustered in two separate groups. The first group, supported by high bootstrap value, included four sequences from three animals (sequences ISS wbUM089 and ISS wbUM60899 deriving from the liver and muscle tissue of the same animal) hunted in two municipalities approximately 80 km apart. The closest phylogenetic relation to this group was the 3i subtype (FJ998008). The second cluster, also supported by high bootstrap value, included eight sequences from seven animals (sequences ISS wbUM103 and ISS wbUM61235 deriving from the same subject) hunted in ATC2 and ATC3 over a 4-month period; four of the sequences were associated to a wild boar detention area.

The wide heterogeneity of the subtypes detected in this study—three subtypes, two clusters not assigned to subtypes (3_uc3 and 3_uc6) and two clusters with no close relation to other available sequences—in a relatively small area (the total surface on Region Umbria is 8.456 km^2^) confirms previous observations on the genetic variability of HEV in wild boar in Italy [36] and, more specifically, in Central Italy [37]. Indeed, as far as wild boar is concerned, circulation of the 3c, 3e, 3f, and of 3_uc3 was reported in a previous study in Umbria [35], and the sole province of Viterbo (440 km^2^), that borders Umbria, hosted the 3a, 3c, and 3f subtypes and a cluster of unassigned sequences, possibly representing a novel subtype [17, 37]. Likewise, occurrence of multiple subtypes (namely 3c, 3e, and 3f) was reported in wild boar in another bordering region, Abruzzo [38]. It should be noted that, given the high variability of genotype 3 of HEV, the continuous revision of its classification into subtypes, and the limited reliability of phylogenetic analysis based on small regions of the genome (in this study, 410 bases within ORF2), the reported assignation to subtypes should be taken with caution and the significance of the unassigned clusters should be further investigated. Indeed, full genome sequencing will be required to provide conclusive phylogenetic assignation of these groups of unassigned strains in comparison to the already recognized G3 subtypes.

## 4. Conclusions

Hepatitis E infection is increasing in industrialized countries, and foodborne transmission is recognized as an important route of transmission for HEV, chiefly in association with consumption of pork and wild boar raw products, including those from domestic production and hunting activities. This study showed that HEV prevalence in wild boar in Umbria is very high (43.6%) and that viral load in wild boar livers may exceed 7 log g.c./g (median concentration: 3.2 log g.c./g). Furthermore, this study highlighted the wide genetic variety of HEV in wild boar population in Umbria, with several different subtypes and unassigned clusters of sequences detected in our survey.

These results indicate that a rigorous surveillance should be enacted on wild boar and/or on derived liver and meat cuts intended for the production of products consumed raw (e.g., dry sausage or salami containing liver) to ensure food safety and to reduce the risk of exposure to HEV for the consumers.

## Figures and Tables

**Table 1:** Primers and probes used for detection and typing of HEV.

Assay	Target	Primer name	Primer sequence	Cycle	Amplicon	References
Real-time RT-qPCR	ORF3	JVHEVF (forward)	5′-GGTGGTTTCTGGGGTGAC-3′	One-step	–	[18, 19]
		JVHEVR (reverse)	5′-AGGGGTTGGTTGGATGAA-3′			
		JVHEVP (probe)	5′-FAM-TGATTCTCAGCCCTTCGC-MGB-3′			

Nested RT-PCR	ORF2	HE044 (forward)	5′-CAAGGHTGGCGYTCKGTTGAGAC-3′	First round	506 bp	[20]
		HE040 (reverse)	5′-CCCTTRTCCTGCTGAGCRTTCTC-3′			
		HE110-2-mod (forward)	5′-GYTCKGTTGAGACCWCBGGBGT-3′	Nested	467 bp	[21, 22]
		HE041 (reverse)	5′-TTMACWGTCRGCTCGCCATTGGC-3′			

**Table 2:** Distribution of the wild boar included in the study, prevalence of hepatitis E virus in liver samples, and median viral load per category.

	No. of tested samples	No. of positive samples	Percentage of positive samples (95% CI)	No. of quantifiable samples	Median viral load (min–max) log_10_ g.c./g of liver
Hunting location					
ATC1 (north)	61	23	37.7% (26.6–50.3)	18	3.78 (1.64–7.35)
ATC2 (southeast)	76	38	50.0% (39.0–61.0)	25	2.58 (1.47–7.32)
ATC3 (southwest)	42	17	40.5% (27.0–55.5)	10	4.10 (1.81–6.65)
Gender					
Male	67	35	52.2% (40.5–63.8)	22	3.05 (1.47–7.35)
Female	47	19	40.4% (27.6–54.7)	13	2.87 (1.72–7.32)
Unknown	65	24	36.9% (26.2–49.1)	18	3.48 (1.82–7.01)
Weight (kg)					
≤50	35	17	48.6% (33.0–64.4)	14	4.49 (1.64–7.35)
>50	76	35	46.1% (35.3–57.2)	21	2.46 (1.47–7.15)
Unknown	68	26	38.2% (27.6–50.1)	18	3.48 (1.82–7.01)
Age (months)					
0–3	4	2	50.0% (15.0–85.0)	1	3.83
4–12	17	10	58.8% (36.0–78.4)	7	5.50 (1.85–7.32)
13–22	24	9	37.5% (21.1–57.4)	4	3.32 (2.40–7.15)
≥23	51	22	43.1% (30.5–56.7)	15	2.37 (1.47–6.39)
Unknown	83	35	42.2% (32.1–52.9)	26	3.26 (1.64–7.35)
Total	179	78	43.6% (36.5–50.9)	53	3.20 (1.47–7.35)

## Data Availability

Genetic data were submitted to GenBank. Other data were available on reasonable request.

## Supplementary Materials

Supplementary Materials Table S1: Prevalence of HEV in wild boar in the different hunting districts.
