# Supplementary material for: High Prevalence and Genetic Heterogeneity of Genotype 3 Hepatitis E Virus in Wild Boar in Umbria, Central Italy
Source: Transbound Emerg Dis. 2023 Jun 30;2023:3126419. doi: 10.1155/2023/3126419 (PMC12017113; doi:10.1155/2023/3126419)
Supplement: Supplementary Materials — Table S1: Prevalence of HEV in wild boar in the different hunting districts. [file 3126419.f1.pdf]

High prevalence and genetic heterogeneity of genotype 3 Hepatitis E virus in wild boar in Umbria, central Italy

Transboundary and Emerging Diseases

Farzad Beikpour, Monica Borghi, Eleonora Scoccia, Teresa Vicenza, Andrea Valiani, Simona Di Pasquale, Silvia Bozza, Barbara Camilloni, Loredana Cozzi, Piero Macellari, Vito Martella, Elisabetta Suffredini, Silvana Farneti

SUPPLEMENTARY MATERIAL

Prevalence of HEV in wild boar within hunting districts

ATC	Administrative district	Number of samples	Number of positive samples	% of positive samples
ATC1	D1	4	3	75%
	D2	6	2	33%
	D3	3	0	0%
	D4	4	1	25%
	D5	9	5	56%
	D6	4	0	0%
	D7	5	0	0%
	D8	4	3	75%
	D9	5	4	80%
	D10	7	3	43%
	D11	6	1	17%
	D12	2	1	50%
	D13	2	0	0%
ATC2	D1	1	0	0%
	D2	8	3	38%
	D3	9	2	22%
	D4	7	1	14%
	D5	4	0	0%
	D6	5	2	40%
	D7	10	6	60%
	D8	17	13	76%
	D9	15	11	73%
ATC3	D1	4	0	0%
	D2	4	2	50%
	D3	7	5	71%
	D4	2	1	50%
	D5	6	4	67%
	D6	2	1	50%
	D7	5	2	40%
	D8	5	0	0%
	D9	7	2	29%
